# Addressing misleading medical information on social media: a scoping review of current interventions

**DOI:** 10.1136/bmjebm-2025-113704

**Published:** 2025-10-05

**Authors:** Emma Grundtvig Gram, Ray Moynihan, Tessa Copp, Patti Shih, Loai Albarqouni, Elie Akl, Courtney Smith, Leah Hardiman, Brooke Nickel

**Affiliations:** 1 Centre and Research Unit for General Practice, University of Copenhagen Department of Public Health, Copenhagen, Denmark; 2 Sydney School of Public Health, Faculty of Medicine and Health, The University of Sydney, Sydney, NSW, Australia; 3 Bond University Faculty of Health Sciences and Medicine, Gold Coast, Queensland, Australia; 4 Australian Centre for Health Engagement, Evidence and Values, School of Social Sciences, University of Wollongong, Wollongong, New South Wales, Australia; 5 Department of Internal Medicine, American University of Beirut, Beirut, Beirut Governorate, Lebanon; 6 Department of Health Research Methods, Evidence, and Impact (HEI), McMaster University, Hamilton, Ontario, Canada; 7 Consumer Representative, NSW, Sydney, Australia

**Keywords:** Evidence-Based Practice, Overdiagnosis, Policy, PUBLIC HEALTH

## Abstract

**Background:**

Misleading information about medical products on social media may cause overuse.

**Objectives:**

Explore interventions targeting the problem of misleading medical information and marketing on social media, with a focus on preventing medical overuse including overdiagnosis.

**Eligibility criteria:**

We included peer-reviewed studies with original data on an intervention targeting misleading medical information on social media and governmental/institutional responses with and without evaluation. We excluded responses relating to COVID-19.

**Sources of evidence:**

four electronic databases: MEDLINE/PubMed, PsycINFO, Academic Search Complete and Web of Science, and searches of grey literature on Google and Google Scholar. Search date: 9 June 2025.

**Data charting:**

We used prespecified data forms populated in duplicate by two reviewers.

**Results:**

We identified 27 peer-reviewed articles and 25 organisational and governmental responses (grey literature). 20 (74%) of the peer-reviewed interventions targeted the consumer to enhance ‘media literacy’, support decision-making or warn about misinformation trends. Approaches included education, such as videos or information materials, to improve detection of misinformation, as well as correcting misinformation and rebutting claims. Only two (7.4%) of the peer-reviewed approaches were sensitive to the problem of medical overuse: a risk-of-deception tool and an informed decision-making service. The grey literature about government and organisational responses chiefly comprised general advertising regulations and other educational resources for consumers to identify and navigate misinformation. The advertising regulations ranged from self-regulatory codes of practice to mandatory regulations, requiring pre-approval of social media marketing material. Most regulations stated advertising should be truthful, presenting both benefits and harms and not be misleading. Most of the grey literature (64%) was sensitive to medical overuse, though none referred explicitly to the problem.

**Conclusions:**

Current efforts to address misleading medical marketing on social media often overlook the critical issue of medical overuse and fail to provide sufficient consumer protections in this rapidly evolving digital landscape of social media, such as the speed of dissemination, reach and the role of third-party advertising. These gaps in research, regulation and practice present significant opportunities to strengthen evidence-based policies and public health responses.

**Trial registration details:**

https://doi.org/10.17605/OSF.IO/2NJSH

**WHAT IS ALREADY KNOWN ON THIS TOPIC**

- Recent evidence suggests that much of the medical information on social media is misleading and may lead to overuse.

**WHAT THIS STUDY ADDS**

- We identified a range of different, potentially useful, interventions for combating misleading medical marketing on social media, ranging from educational efforts to improve detection of misinformation to advertising legislation.

**HOW THIS STUDY MIGHT AFFECT RESEARCH, PRACTICE OR POLICY**

- Continued presence of misinformation on social media is testament to the fact that regulations are inadequate in responding to the unique and evolving setting of social media, including speed of spread, reach and third-party advertising.
- We need multidisciplinary collaborations and effective means to enforce or implement interventions that account for the diverse nature of advertising on social media, specific to the issue of overuse.

## Introduction

With the rise of social media, medical and health information has become more accessible, yet this accessibility comes with unique challenges.^1 2^ Social media platforms are designed to maximise user engagement by promoting content that captures attention, often favouring information with sensational or eye-catching features.^3^ Consequently, medical information that is misleading—whether it includes false, unverified, unbalanced or exaggerated claims—is often more likely to reach larger audiences.^4–6^ This challenge is compounded by social media’s rapid and widespread dissemination of information without verification or authentication processes.

While enhanced access to information can be seen as a positive and democratising advance for consumers, misleading medical information is particularly concerning because of its potential impact on health decisions.^7^ Decisions about health and medicine are deeply personal, affected by fear, societal norms and resources. However, commercial interests frequently exploit this dynamic to promote medical products, leveraging platform algorithms to target specific groups with personalised ads.^8–11^ Health-related content, when inaccurate, may lead consumers to make uninformed choices, delay essential treatments or even pursue harmful actions that can lead to overtesting, overdiagnosis and overtreatment.^12 13^ A recent study of almost 1000 posts on TikTok and Instagram about medical tests found the overwhelming majority of posts were misleading, failing to mention potential harms of the tests, including overdiagnosis and overuse.^14^ This strengthens the evidence base about medical misinformation on social media.^3 4 15–17^ In response, it is time to research and develop effective strategies to address misinformation.^18^

Researchers have suggested platform-targeted measures to tackle misinformation, including fact-checking, removal of misinformation, correction and warning labels.^19 20^ A review on combatting conspiracy theories has identified inoculation messaging and media literacy interventions as promising countermeasures.^21^ However, the changing and ambiguous nature of misleading or misinformation is outpacing evidence and WHO is currently campaigning against health-related misinformation.

This scoping review aims to identify interventions and strategies that address misleading medical information on social media. A secondary objective is to focus on how these proposed strategies deal with preventing overuse of medical resources. In the face of the seemingly overwhelming problem of misleading medical information, this review identifies responses to this challenge, for policymakers, health professionals, researchers and citizens interested in safe and sustainable use of medical resources.

## Methods

This scoping review is conducted in accordance with Joanna Briggs Institute standards and reported in accordance with the Preferred Reporting Items for Systematic Reviews and Meta-Analyses (PRISMA) Scoping Review extension.^22 23^ A protocol was developed and published prospectively on Open Science Forum.^24^

### Eligibility criteria

We included both peer-reviewed citations and grey literature as these are seen as equally important sources for combating misleading information; for example, grey literature such as regulations can be powerful tools for controlling advertising. As described in detail in our protocol,^24^ eligibility was assessed based on three criteria outlined below and was not restricted by language.

1. Responses/solutions to misleading medical information on social media: we included responses to misleading medical information, including misinformation and disinformation.^6^ Drawing on the work of El Mikati and colleagues,^6^ for the purposes of this review, we define misinformation as ‘false information that may or may not be intentional’ and disinformation as ‘intentional dissemination of false information’. We use ‘misleading’ to describe medical information giving a false impression—such as exaggerating benefits and/or ignoring harms—whether disseminated intentionally or not. We excluded generic responses not specific to medicine, as medical and health-related misinformation often has distinct financial motives and impacts compared with other topics (eg, political or environmental misinformation). COVID-19-specific responses were also excluded due to extensive existing reviews and the distinct event-related features of a global pandemic.^19 25–27^
2. Social media: we included responses to misleading medical information, and related terms, on social media.^28^
3. Citations from academic databases had to be peer-reviewed and include original data. From grey literature searches, we included legal reports and institutional or governmental actions, including those without original data. As institutional and government reports and strategies often rely on evidence synthesis, inclusion of original data was not a requirement for grey literature.

### Search strategy

We performed the search strategy in the following electronic databases: MEDLINE/PubMed, APA PsycINFO, Academic Search Complete and Web of Science. The search strategy was initially developed for PubMed/MEDLINE and then adapted for other databases, with adjustments to controlled vocabulary and subject headings as needed.^24^ This strategy was created in collaboration with two information specialists from the University of Copenhagen and Bond University, respectively (online supplemental file 1).

10.1136/bmjebm-2025-113704.supp1 Supplementary data

Grey literature searches were performed in Google and Google Scholar, websites of influential governments and health organisations and grey literature databases.^24^ Key terms were searched in each platform or website noting the number of hits. At least the first 100 hits were reviewed, relying on relevance at the top of the search. Screening continued in case of relevance (in six cases more than 100 hits were screened). The stopping rule was based on relevance, for example, searching Google, we reviewed 240 hits and continued until at least two pages did not provide any hits that were related to the topic.

The academic database searches were performed on 15 August 2024 and grey literature was searched on 19 August 2024. Searches were updated on 9 June 2025. The original search was performed in Australia and the updated search in Denmark.

### Selection process

Two authors independently assessed the eligibility of title, abstract and full-text level articles in Covidence. Disagreements were resolved through discussion with involvement of a third author in case of non-consensus. Prior to formal selection, two authors screened a selection of citations until agreement was >75%.^22^ Inter-rater reliability rates of agreement were 97.65% on title and abstract level and 81.01% on full text level.

We screened reference lists of identified relevant reviews and included studies.

We contacted all corresponding authors of included citations for input on relevant organisational or governmental strategies, specifically inquiring about responses from their respective countries.

As per protocol, we did not pose any language restrictions; if non-English papers were identified, the title was translated using online translating services.^24^ If the title seemed relevant the full citation was then reviewed by a native speaker. This only became necessary for Chinese citations.

### Data extraction

Data charting was performed in duplicate by two reviewers. Calibrated forms had been checked and piloted by four members of the review team before their use. Official data extraction started when pilot extractions had 75% or greater agreement.^29^ We extracted data about the type of response and if these were sensitive to the problem of medical overuse. We judged sensitivity to overuse if the interventions specifically included parts that were targeted at this problem or if they discussed the development of the intervention as a response to the problem. We defined overuse as a service or type of care that causes harm with little to no accompanying benefit including related terms such as medicalisation, low-value care, overdiagnosis and overtreatment.^30^

### Data synthesis

Data was extracted using a comprehensive list of predetermined items. These items were developed by the author group which has expertise spanning medicine, public and global health, journalism, sociology and consumer insights. The tabularised data was reviewed and discussed within the author group, whereafter EGG drafted an analysis including reporting frequencies, which was then discussed within the author group. As per protocol, the responses and key characteristics were mapped based on an inductive content analysis.^31^ First, the data was extracted from all citations, and then based on extracted data including description of intervention, aims, target group, the responses were openly coded allocating characteristics into overall categories.^31^ Categorisations are presented in a figure with visualisation of codes as well as in text.

Since organisational and governmental strategies, including regulation, do not necessarily present an evaluation of the intervention, these were reported in a separate table to allow for different key features of the types of responses. Some organisations presented multiple strategies, if the organisational responses were similar or guidance for actors were based on the same law, these were presented collated in the analysis. All URLs (uniform resource locators) for collated responses are provided.

## Results

### Selection of citations

**Figure 1:**
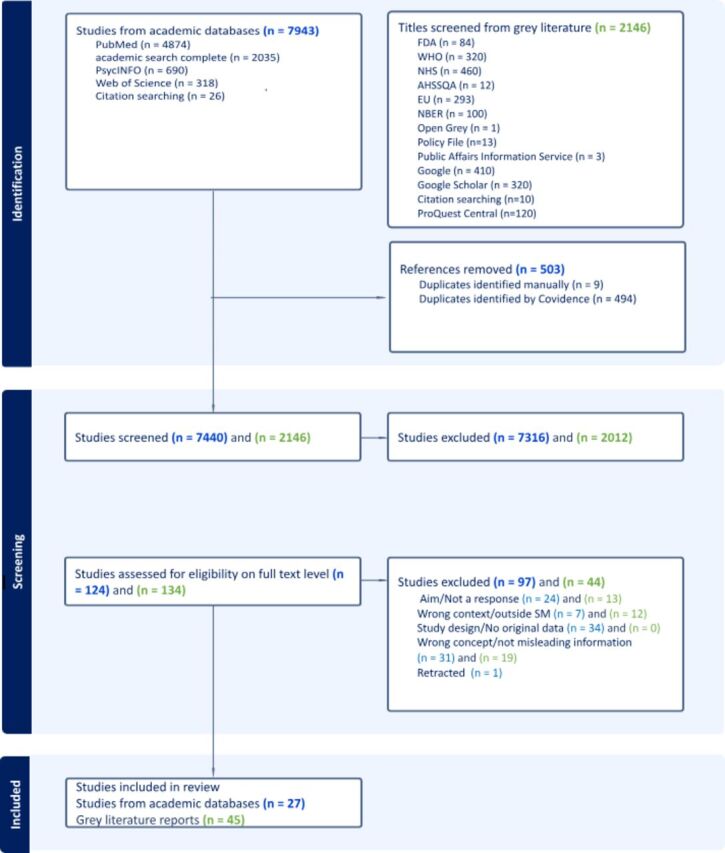
Preferred Reporting Items for Systematic Reviews and Meta-Analyses flow chart.

We included 27 peer-reviewed articles and 45 grey literature reports. The selection process is visualised in figure 1.

The majority of the excluded peer-reviewed literature at full-text level was due to the lack of original data (figure 1). For grey literature, citations were primarily excluded because they did not pertain specifically to health.

### Characteristics and results of included peer-reviewed literature

Table 1 presents the characteristics of included peer-reviewed literature. Supporting evidence on definitions, study design, funding and conflicts of interest is presented in online supplemental file 2.

**Table 1:** Characteristics of interventions responding to misleading medical information on social media; peer-reviewed literature

Author, year, country*	Aim of intervention	Description of intervention	Targeted	Target group	Design/development	Target outcomes	Overuse†
Alsaad and AlDossary, 2024, Saudi Arabia^32^	Help consumers detect misinformation	Educational video intervention based on WHO’s advice on how to navigate and identify misinformation. Concepts included analysing facts, checking links, assessing photos and videos	Health information on WhatsApp	Consumers	Inoculation and the message interpretation process theory	Ability to identify and knowledge about misinformation	No
Au *et al*, 2021, China^50^	Prevent spread	An experiment using financial incentives and legislation, punishing misinformation spread	Healthcare misinformation	Consumers	Evidence synthesis and expert Delphi	Intention to share misinformation	No
Bode and Vraga, 2017, USA 40	Correct misinformation	An intervention on correction techniques: peer correction and algorithmic correction through the related stories function on Facebook	Misinformation on Zika virus on Facebook	Consumers	Evidence synthesis	Misperceptions and perceived credibility	No
Braga *et al*, 2025, Portugal^97^	Monitor and identify misinformation	An online social listening intervention to identify emerging misinformation trends	Misinformation about non-communicable diseases on Twitter	Health workers, public	Evidence synthesis, expert consultation and analysis of social media data	Performance of keywords to detect misinformation	No
Brooks *et al*, 2023, USA^57^	Address health misinformation	A multidisciplinary virtual centre aiming to detect misinformation on social media using platform algorithms, evidence reviews and public communication	Infodemics across a range of platforms and blogs	Consumer, health workers, ministries	Interdisciplinary expert groups and social media analysis	Ability to detect misinformation	No
Byrne *et al*, 2024, Ireland^37^	To support informed decision-making	iHealthFacts solicits questions from the public about health claims, conducts evidence reviews and presents the findings in an accessible way	Unreliable health information	Consumers	Evidence synthesis, public involvement and organisation collaboration	Critical thinking, informed-decision making	Yes
Di Sotto and Viviani, 2022, Multiple^54^	Help platforms detect misinformation	An algorithm for detection of misinformation using machine learning	Health misinformation on Twitter	Technology	Social media analysis and evidence synthesis	N/A	No
Elhariry *et al*, 2024, India and UK^33^	Train medical students to create evidence-based information for social media dissemination	Developing evidence-based education videos for social media	Misinformation about PCOS and thyroid disorders across social media	Public	Evidence synthesis and expert and patient panels	Outreach and audience engagement on social media and participant experience	No
Erim *et al*, 2025, Nigeria^38^	Train participants and communities in identifying and addressing health information	Establishing a health misinformation fellowship programme training participants to combat health misinformation through community-based approaches	Health misinformation	Public	Stakeholder and community engagement	Reach and impact of the activities and the confidence in identifying and mitigating misinformation	No
Fridman *et al*, 2025, USA^55^	Identify misinformation	Developing a framework of linguistic characteristics of misinformation that could be easily observed by users to help them identify and flag misinformation on social media	Misinformation about unproven cancer treatments on Twitter	Consumers, algorithms	Evidence synthesis and social media data mining	Performance of linguistic characteristics/prediction of misinformation labels	No
Garrett *et al*, 2019, Canada^53^	Help healthcare professionals educate public on health scams	A risk-of-deception tool to detect potential internet health scams/assess the likelihood of deceptive content	Health scams	Health workers	Evidence synthesis and Delphi approach, with health professionals	Deceptiveness of posts	Yes
Gesser-Edelsburg *et al*, 2018, Israel^47^	Correct misinformation on measles vaccine	An intervention on corrective information sensitive to the emotional domain of health information (voicing health concerns)	Health misinformation on Facebook	Consumers	Literature	Self-efficacy, reliability, behavioural intentions	No
Ku *et al*, 2025, China^41^	Correct misinformation	Using logic or fact-based corrections combined with hashtags (inclusivity vs health literacy framings)	Misinformation about Mpox on Instagram	Consumers	Amended from Vraga *et al* 98	Misperceptions, attitudes, correction sharing likelihood	No
Lazard and Queen, 2025, USA^42^	Encourage intervening and prevent spread	Using social cue prompts (content warning labels) to encourage flagging of misinformation to reduce sharing and increase post removal by platforms	Misinformation about cancer treatment	Consumers	Evidence synthesis	Willingness to intervene, sharing intentions and perceived responsibility, empathy and acceptability	No
Leininger *et al*, 2022, USA^39^	Educate public and infodemic management	A set of core communication principles: listening, empathy, engage, transparency; communication strategies including prioritising graphic design, using examples, analogies and narratives	Health misinformation	Consumers	Expert panels	N/A	No
McPhedran *et al*, 2023, UK^34^	Decrease engagement	An educational inoculation intervention with ‘threat’ and ‘pre-bunking’ components and information about dangers of misinformation, resources for identification and a source reliability checklist	Health misinformation	Consumers	Inoculation theory	Engagement with misinformation content	No
Mende *et al*, 2023, USA^35^	Warn consumers	A conceptual model that offers a lens through which to evaluate warning labels and disclosures	Misinformation and disinformation	Consumers	Evidence synthesis	Negative impacts of infodemics	No
Nazarnia *et al*, 2023, Iran^36^	Improve media health literacy	A mobile-based educational intervention on ‘media health literacy’ across multiple domains	Health misinformation	Consumers	Evidence synthesis, health literacy theory and a team of app designers	Media health literacy	No
Ozturk *et al*, 2015, USA^51^	Reduce spread	An intervention on counter statements and warnings accompanying misinformation	Health rumours on Twitter-like platforms	Consumers	Evidence synthesis	Intention to share misinformation	No
Sun and Pan, 2025, China^43^	Reduce spread	An intervention testing ways of correction (simple rebuttal and factual elaboration) and correction source (AI and human fact-checking)	Health misinformation on Weibo	Consumers	Evidence synthesis and Medical Fact-Checks on Snopes.com	Intention to share misinformation and perceived credibility of source	No
Upadhyay *et al*, 2023, Italy^56^	Help platforms detect misinformation	A deep-learning model for automatic detection from structural and content-based characteristics, in context of a multilayer architecture	Health misinformation	Technology	Machine learning using source, content and design features	Genuineness of health information	No
Vraga and Bode, 2017, USA^44^	Correct misperceptions	An intervention with corrections posted by either social media users or governmental institutions	Misinformation about Zika virus on Twitter	Consumers	Evidence synthesis	Misperceptions credibility trustworthiness	No
Vraga and Bode, 2018, USA^45^	Correct misperceptions	Social correction feedback from peers testing both the type of correction and the platform	Misinformation about Zika virus on Facebook and Twitter	Consumers	Evidence synthesis	Misperceptions credibility/trustworthiness	No
Vraga *et al*, 2019, USA^46^	Correct misinformation	An intervention combining exposure to weak forms of misinformation after exposure to the original misinformation (inoculation) and corrections	Misinformation about HPV‡ vaccine on Twitter	Consumers	Inoculation theory and evidence-synthesis	Credibility misperceptions	No
Vraga *et al*, 2022a, USA^48^	Correct misinformation, enhance news literacy	Two solutions pairing news literacy messages with corrective responses to health misinformation	Misinformation on Twitter	Consumers	Evidence synthesis	Credibility misperceptions perceived news literacy	No
Vraga *et al*, 2022b, USA^52^	Correct misinformation about skin cancer	An intervention combining peer correction and news literacy video messages that warn audiences about misleading content on social media and the need to be sceptical (educational inoculation)	Misinformation about skin cancer on Facebook	Consumers	Evidence synthesis and social media observations	Misperceptions Intention to wear sunscreen	No
Vraga and Bode, 2025, USA^49^	Mitigating impact of exposure to misinformation	A correction intervention using truth signals (whether the person posting the story says it is true, whether the replies to the story say it is true, or whether the story itself is actually true)	Health misinformation	Consumers	Evidence synthesis and Medical Fact-Checks on Snopes.com	Perceptions of story veracity, attitude alignment with the content and related behavioural intentions	No

*Country of study conduct.

†Sensitive to the problems of overuse.

‡

§If no social media platform is mentioned, the intervention target social media generically.

AI, Artificial Intelligence; HPV, Human Papilloma Virus; PCOS, Polycystic Ovary Syndrome.

20 out of 27 (74%) interventions presented interventions targeted at the consumer. These consumer-targeted interventions were primarily targeting misinformation through educational means such as videos or information material^32–36^ or through evidence reviews and communication.^37–39^ These interventions aimed to improve health media literacy, support decision-making or warn consumers about misinformation trends. Other consumer-targeted interventions aimed to correct misinformation, rebutting claims either to lower credibility or address misperceptions. These interventions included corrections introduced by peers,^40–45^ pre-exposing consumers to truthful information (inoculation),^46^ accounting for fear of disease,^47^ educational messages^48^ and truth signals.^49^ Others aimed to reduce the spread of misleading information through financial incentives and punishment^50^ or warning labels.^51 52^

One intervention targeted health professionals, presenting a tool to help assess the likelihood that specific social media health content is deceptive.^53^ There were algorithmic responses to detect misinformation using machine learning for content moderation on platforms.^54–56^ One multi-targeted and multidisciplinary intervention used automatic artificial intelligence (AI)-generated alerts about misinformation, reviewed and communicated evidence and proposed these were circulated by government ministries.^57^ Most responses targeted medical misinformation in general, while a few targeted misinformation specifically about Zika virus,^40 44 45^ human papillomavirus vaccines,^46^ skin cancer^52^ or measles vaccine.^47^ Identified responses relied primarily on literature reviews and evidence synthesis for developing interventions.

### Characteristics and results of included grey literature

Table 2 presents the characteristics of the included grey literature. We identified 45 independent websites which were collated into 25 responses.

**Table 2:** Characteristics of interventions responding to misleading medical information on social media; organisational and governmental responses

Organisation	Country	Year	Description of response	Target group	Overuse*
Therapeutic Goods Administration, Department of Health and Aged Care^99–101^	Australia	2023	Regulation for industry and social media influencers on advertising therapeutic goods	Industry and social media influencers	Yes
The Australian Health Practitioner Regulation Agency^60^	Australia	2020	Industry guidance clarifying legal responsibilities in advertising regulated health services	Health workers	Yes
Therapeutic Goods Administration, Department of Health and Aged Care^58^	Australia	2020	Online resource on how to spot a dodgy health product ad	Consumers	Yes
The Digital Industry Group and the Australian Communications and Media Authority^66 67 102^	Australia	2024	The code of practice conduct and a bill supporting self-regulation code on disinformation and misinformation on social media platforms	Platforms	No
Health Canada^103 104^	Canada	2002	Regulation on advertising health products	Industry	Yes
Health Canada, Government of Canada^65^	Canada	2020	Proactively monitoring drug and device marketing to enforce advertising regulation, request cessation of illegal activity, use regulatory sanctions and partnerships to encourage using voluntary code	Industry, health workers, organisations	Yes
Health Canada, Government of Canada^59^	Canada	2019	Online resource with information about misleading marketing and current trends to beware of, industry guidance and reporting tool	Consumer, health workers	Yes
Chinese Government^63 64^	China	2015	Regulation for industry on audio-visual advertising of health foods and medical devices on social media	Industry	Yes
Danish Ministry of Health^105–107^	Denmark	2013	Regulation on advertising healthcare products, medical devices and drug	Industry/advertisers	Yes
European Parliament^108–110^	Europe	2025	Overview of actions designed to strengthen cooperation between the EU member states including exchanging information on misinformation and orchestrate coordinated responses. Upcoming initiatives also include the Consumer Agenda 2025–2030 and the Digital Fairness Act to strengthen consumer protection against harmful online practices, complementing the existing EU digital regulations	All sectors of society	Yes
European Commission^111^	Europe	2021	The EUDAMED database providing an overview of the lifecycle of medical devices available in European Union, to improve access to credible information and enhance coordination across governments	Industry, governments	No
European Commission^112–114^	Europe	1992/2006	A regulatory regime on management and advertising of prescription drugs and medical devices to ensure safety while supporting innovation and competition	Industry	No
European Commission^68^	Europe	2018	Code of practice on disinformation promoting self-regulation	Platforms and industry/advertisers	No
Maharashtra Medical Council, Mumbai^115 116^	India	2024	The Medical Council is taking action against misleading advertising on social media by registered practitioners	Health workers	Yes
Dubai Health Authority^117^	UAE	2022	Regulation on advertising medical products on social media	Health workers	Yes
Medicines and Healthcare products Regulatory Agency^118^	UK	2022	Regulation on advertising medicines and health products	Industry	Yes
US Department of Health and Human Services^119–121^	USA	2024	Regulatory guidance for all sectors of society including the US Surgeon General’s Advisory	All sectors of society	No
National Academy of Medicine^122^	USA	2021	Online resource with guidance for identification of credible health information on social media	Platforms and consumers	No
Food and Drug Administration^123^	USA	2023	Online information about health scams, identification of health scams and how consumers can protect themselves	Consumers	Yes
Food and Drug Administration^124–126^	USA	2025	The Bad Ad Programme reviews prescription drug ads for misleading content, risk-based surveillance with a growing focus on social media influencers and telehealth companies. A new bill, the ‘Protecting Patients from Deceptive Drug Ads Act’ (introduced February 2025), aims to expand FDA oversight to include telehealth firms, pending its passage into law	Industry	Yes
Food and Drug Administration^61^	USA	2014	Industry guidance clarifying legal responsibilities in third-party misinformation about drugs and medical devices on social media	Industry	Yes
Food and Drug Administration^62^	USA	2024	Industry guidance clarifying legal responsibilities presenting risk and benefit information for drugs and devices on social media	Industry	Yes
WHO^127–130^		2022 (up-dated 2024)	WHO collaborates with tech companies and expert panels to identify and flag health misinformation, helping prevent its spread and work with policy teams on guidelines for content providers, including efforts with YouTube, Google, Facebook and NewsGuard to remove misinformation and promote science-based health information	Consumers, technology, industry	No
WHO and European Parliament^131^		2022	Make recommendations on collaborative action across sectors to better protect people from misinformation and mitigate the harms	Public, industry, regulators	No
WHO^132^		2020	WHO facilitates the Fides network to unite health professionals and help them deliver evidence-based recommendations for health targeting prevalent misinformation on social media	Consumer and health workers	No

*Sensitive to the problems of overuse.

EU, European Union; EUDAMED, European database on medical devices ; FDA, Food and Drug Administration.

16/25 (64%) were targeted at the industry or advertisers, including advertising regulations or codes of practice, 8/25 (32%) were educational resources for consumers, industry and health professionals and the rest were collaborations or programmes to monitor advertising activities (table 2).

Educational responses included online resources for consumers to detect and avoid online health scams^58 59^ and resources for health professionals to educate the public or how to comply with regulation.^60–62^ The identified regulations were employed to protect the public from misinformation and potential downstream harms. Identified regulations varied across countries but almost all—except the USA—forbid the advertising of prescription drugs to the public. The identified legislation did not have separate rules for advertising on social media, but encompassed social media along with other forms of media, such as television, radio and billboards. China had regulations specifically for online advertising including that medical or health advertising should receive prior approval from health authorities.^63 64^ All included regulations stated that content of medical advertising should be truthful, should present both benefits and harms and not be misleading.

Liability for content is different across the included regulations; in Australia, the USA and China, the advertiser is responsible for the accuracy of content, which means that third-party distributors can be liable. In Denmark and the rest of the European Union (EU), it is primarily the company sponsoring the advertiser who is responsible for the compliance of advertisement. In most of the included regulatory regimes, there are no oversight programmes in place besides so-called ‘claim-based sites’, where consumers and the public can flag potential non-compliant advertising to the government, who then choose whether to investigate and/or pursue legal enforcement. Health Canada has a monitoring programme that oversees advertising activities on social media.^65^ The monitoring programme targets illegal advertising by enforcing compliance, applying sanctions and recommending charges when necessary and emphasises education through optional preclearance, accrediting preclearance agencies and interest-holder training.^65^ We identified self-regulations and voluntary codes for Canada, Australia and the EU.^66–70^ These codes involve voluntary preclearance processes for advertisers, commitments of social media platforms to perform content moderation, disclose paid advertising and make use of fact-checking services.

### Perspectives on medical overuse

The vast majority of the peer-reviewed responses focused on how to avoid misinformation that may cause delays in seeking appropriate healthcare or foregoing relevant services. In other words, they were framed in some way around preventing information which may drive ‘underuse’. Only 2 of the 27 peer-reviewed responses (7.4%) were considered sensitive in some way to the problems of medical overuse^37 53^ (table 1), although neither explicitly mentioned overuse. For example, Byrne and colleagues present an educational tool to encourage critical thinking, specifically about unproven and potentially harmful medical treatments^37^ and Garret and colleagues describe one for detecting health scams defined as ‘*…* products (that) are sold based on exaggerated claims or falsehoods—and the use of mass media to facilitate scams (…)’ to avoid the public being misled to take up harmful or non-evidence-based services and treatments.^53^

Of the organisational or governmental responses analysed, none of them explicitly referred to medical overuse or related terms such as overdiagnosis, but 16 out of 25 (64%) demonstrated some form of sensitivity to these problems (table 2). These responses included contributions from the FDA (Food and Drug Administration, USA), TGA (Therapeutic Goods Administration, Australia), Health Canada, as well as authorities in Dubai, China and Denmark, all explicitly emphasising the objective to protect patients and the public by preventing engagement with potentially harmful services. In contrast, the WHO and EU primarily focused on mitigating harm caused by patients forgoing effective treatments due to misinformation and did not address the risk of misinformation leading to the uptake of harmful or unnecessary services or general overuse.

### Thematic map of responses

Informed by existing frameworks,^71 72^ we iteratively categorised identified responses. We identified four distinct categories: educational, debunking, algorithmic and regulatory responses. The educational responses were interventions applying information or learning targeted at improving detection, preventing spread, as well as improved awareness, literacy and decision-making (n=18). Debunking responses exposed misinformation through corrections or warning labels with the aim to prevent their spread and improve misperceptions (n=13). Algorithmic solutions included development or employment of a set of computational instructions for platforms to help detect misinformation (n=3). Regulatory responses were legally-binding laws or codes made and maintained by authorities (n=17). These sought to protect citizens by upholding ethical standards, safety, public health, guidance on compliant advertising and monitoring. As can be seen in the figure, these categorisations were not clear-cut and characteristics of interventions were overlapping.

**Figure 2:**
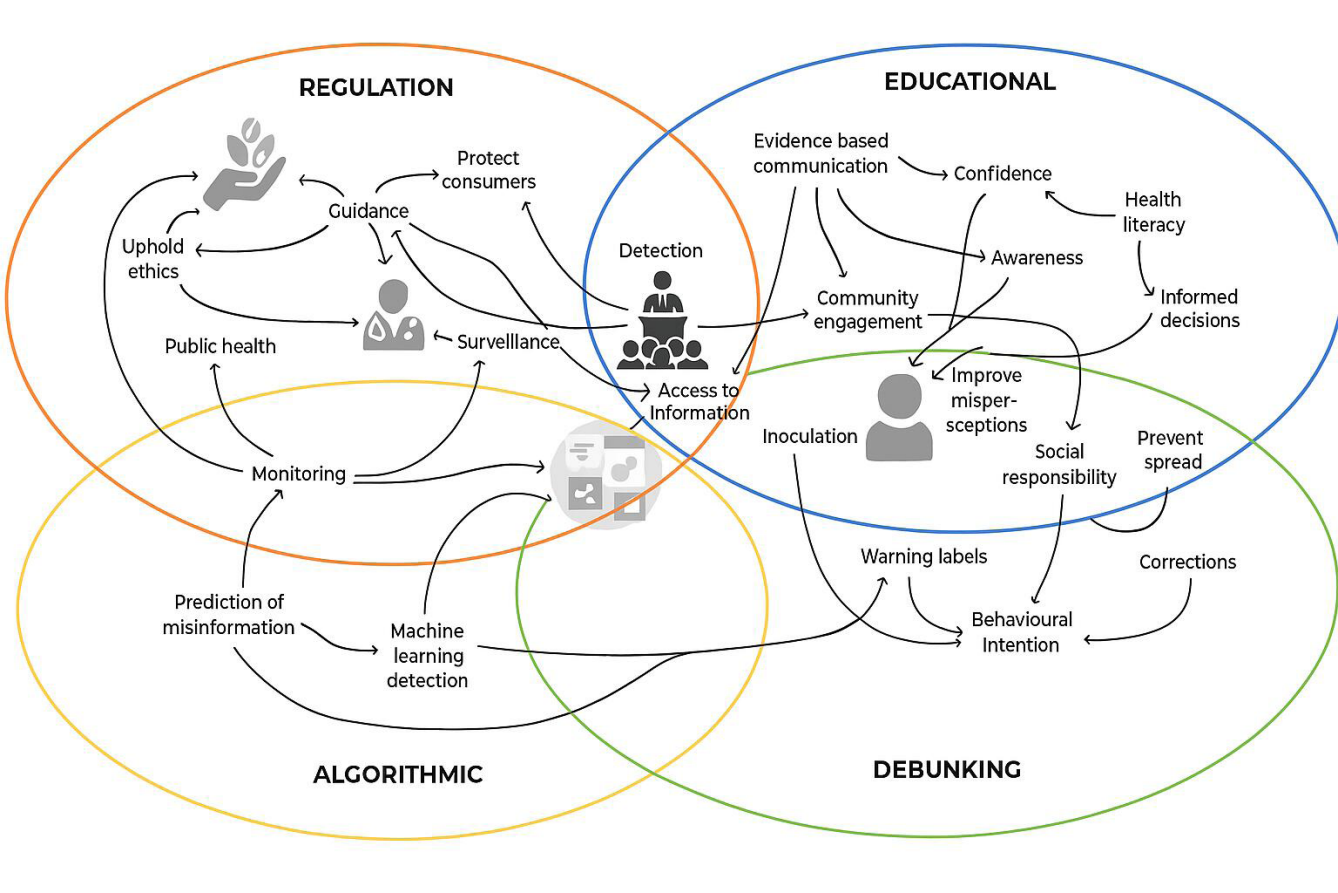
Categorisation of responses with accompanying aims and levels they target. AHSSAQA, Australian Health Service Safety and Quality Accreditation; EU, European Union;FDA, Food and Drug Administration; NBER, National Bureau of Economic Research; NHS, National Health Service; SM, Social Media; WHO, World Health Organisation.

The categorisation displayed in figure 2 communicates the interconnectedness of public health goals, intervention strategies and their applications at different levels of society.

## Discussion

Through a scoping review of peer-reviewed and grey literature, we identified four categories of responses to misleading health information on social media: educational, debunking, algorithms and regulation. Solutions proposed in peer-reviewed literature were primarily educational and debunking targeted at consumers, aiming to improve awareness and ability to detect misinformation through public information material, corrections and warning labels. Among grey literature, we identified advertising regulations which all required truthful, balanced content while mostly banning public advertising of prescription drugs. However, few regimes had social media specific regulations or monitoring programmes in place to account for the special features of social media advertisement and to oversee the large and growing amount of advertisement. Only 7.4% of peer-reviewed interventions were sensitive to problems related to medical overuse, compared with 64% of organisational responses, with most of these primarily aiming to prevent the uptake of harmful services.

Our review has important limitations. The inter-reliability in the screening process was 97.65% on title-abstract level and 81.01% on full-text level pointing to some degree of selection inconsistency. We have employed an inclusive approach meaning opting for inclusion if in doubt combined with poor reporting (eg, framing as a health/medical problem but using political misinformation examples to develop strategy), both contributing to low inter-reliability. While some national regulations on health advertising were included, our approach was not exhaustive as we did not seek out all national laws. Additionally, as per standard scoping review methodology, we did not assess the effectiveness or quality of the included interventions and thus cannot conclude which interventions are more effective. We excluded expert opinions and academic literature without original data, which might offer valuable insights and potential solutions. We did, however, analyse opinion pieces identified in our systematic search, summarised their recommendations and considered their insights in this discussion.

The majority of identified responses focused on consumer-targeted interventions relying on the consumer’s capacities; for example, to be more aware of misinformation, improve detection or change behaviour. This is similar to other reviews mapping countermeasures to fake information or conspiracy theories on social media.^19 21^ However, other recent reviews^73 74^ and experts are calling for collaboration including multi-targeted and interdisciplinary approaches.^75–79^ Our included studies primarily rely on literature reviews and evidence synthesis to design and develop interventions to combat misinformation and might be lacking important perspectives from consumers and organisations. Experts suggest that relying on the capacities of the consumer alone to try and combat misinformation might be overly simplifying extremely complex problems, and is not commensurate with the challenges posed by the highly misleading information and advertising currently flooding social media.^73^ Further, consumer-targeted interventions such as educational responses might require large amounts of resources and will likely only reach people with higher socioeconomic status and health literacy. Combined with increased susceptibility to misinformation among people with lower socioeconomic status, such interventions might be ineffective or produce further inequity in health.^80^ Further, spending resources on improving health literacy in the context of the promotion of tests or treatments that do not have evidence of benefits and thus should not be advertised in the first place seems non-sensical. Researchers, organisations, editors and grant-providers should work together to develop and communicate efficient, evidence-based strategies that resonate with policymakers and stakeholders.^71 81^ These should focus on testing larger-scale collaborative, multidisciplinary and system-level interventions to combat misleading information and marketing on social media rather than focusing on responses that posit responsibility solely with individual consumers. Further, experts are pointing to the need for interventions targeting the problems surrounding celebrity endorsement, pervasive use of anecdotal evidence, influencer marketing and peer-to-peer advertising.^82–86^

We found that almost all of the peer-reviewed interventions focused primarily on addressing the harms of medical ‘underuse’, while being largely insensitive to the challenges posed by overuse and overdiagnosis. This reflects the wider fact that concerns and debates about medical misleading information on social media have to date focused almost exclusively on misinformation which may deter or delay appropriate care, rather than misinformation designed to drive overuse of unnecessary care—often in the context of conflicts of interest. In our view, this has created an evidence gap, with very limited exploration of social media promotion which may be driving overdiagnosis and overuse. The problem of overuse, and associated harm and waste, is increasingly recognised as a major challenge taking resources away from addressing underuse of appropriate care.^87^ ^88^ Practitioners struggle to deliver evidence-based and compassionate care to patients who need it within the time available to them.^89^ Moreover, overuse contributes to environmental harms, including the growing carbon footprint of healthcare.^90^ Thus, we argue there is a need for more consideration of the harms of overuse when designing and evaluating the impact of interventions aimed at mitigating misleading medical information.

The included national advertising legislation, which is far from an exhaustive list, shows that regulation is in place to target misleading advertising and points towards a greater awareness of the problem associated with overuse and industry conflicts of interest. However, there seems to be less engagement within national regulations, with the unique and growing problems posed by social media such as speed of spread, reach and third-party advertising. Current engagement with these problems might offer inspiration for new regulatory strategies effective in combating the harms from misleading medical advertising on social media. For example, Denmark is currently enforcing an approach where all influencers are to be considered independent companies, and where all claims, sponsored or not, should comply with the National advertisement code.^91^ Concurrently, China has banned celebrities from publicly endorsing or advertising health products,^63 64^ although allegedly enforced to promote socialist values.^92^ Regulators may benefit from employing regulations that are sensitive to social media, as seen in other areas such as cosmetic surgery.^93^ Since we did not assess the effectiveness of the included interventions, we are hesitant to give advice, but these examples might provide inspiration for future directions.

Studies show that health misinformation is booming on social media, even in the context of current regulations.^4 15–17^ Although we did not map the entire regulatory landscape, it is clear that current regulatory frameworks are inadequate and that the dissemination of medical misinformation has outpaced the ability of governments and regulatory bodies to create, develop and enforce effective countermeasures.^14^ Health Canada has established a dedicated effort to oversee and enforce advertising laws on social media, which might be one example of a positive way forward. There seems to be a willingness to update current regulatory regimes. For example, New Zealand was previously one of the only nations allowing direct-to-consumer advertising of prescription drugs, but it will be revisited in a new bill in 2025.^94^ Closely analysing different regulatory regimes would be helpful to inform and inspire guidelines and policies and valuable for the work of policymakers who are engaged in designing or evaluating responses. Further, evaluating the effectiveness and compliance with these responses would be helpful for directing future resources.^95 96^

Our searches were performed in Australia and Denmark, which ought to have little to no effect on searches in academic databases; this might impact results from grey literature searches due to geographical filters and algorithms, ensuring broader representativeness. We did not pose any language restrictions to the eligibility criteria but performed searches using English words, potentially down prioritising non-English pages, which might be the reason for the lacking perspectives from the Global South.

### Conclusion

Current efforts to address misleading medical marketing on social media commonly overlook critical aspects related to the unique challenges posed by social media sharing mechanisms and medical overuse. Future strategies could benefit from interdisciplinary and structural interventions that consider the potential harms of overuse and focus on a comprehensive evaluation of social media advertising practices including celebrity endorsement, influencer marketing and rapid sharing of content. These insights could provide valuable guidance for policymakers aiming to regulate and mitigate the impact of misleading health marketing on social media.

## Data Availability

All data relevant to the study are included in the article or uploaded as supplementary information. All data is available in the article or supplementary files.
