# Supplementary material for: Addressing misleading medical information on social media: a scoping review of current interventions
Source: BMJ Evid Based Med. 2025 Oct 5;30(6):420–33. doi: 10.1136/bmjebm-2025-113704 (PMC12703284; doi:10.1136/bmjebm-2025-113704)
Supplement: Supplementary data [file bmjebm-30-6-s001.pdf]

Supplementary File 1: Search Strategy

Each block is combined with the logic term: AND

Search block	Responses	Misleading	Medical	Social media
**MeSH headings**	"Social Control, Formal"<br>"Government Regulation"<br>"Medical Device Legislation"<br>"Drug and Narcotic Control"<br>"International Health Regulations"	"Health Literacy"<br>"Communication"	Not included in Pubmed.	"Social media"<br>"Media exposure"
**Text-words**	Regulation*<br>recommendation*<br>legislation*<br>polic*<br>response*<br>effort*<br>"preventive measure*"<br>"combat*"<br>"infodemic management"<br>debunk*<br>solution*	"False information"<br>"incorrect information"<br>"Inaccurate information"<br>"misinformation"<br>"disinformation"<br>Infodem*<br>"false science"<br>"misleading information"<br>"fake news"<br>malinformation<br>"health washing"<br>"unfair information"<br>"health literacy"<br>"digital health literacy"<br>"information literacy"	Medical<br>Health<br>Medicine<br>Test<br>Diagnos*<br>Treatment*<br>Therap*	TikTok<br>Instagram<br>Facebook<br>Twitter / X<br>Youtube<br>Social media<br>Snapchat
**Search (each block combined with logic term: AND)**	(("social control, formal"[MeSH Terms] OR "Government Regulation"[MeSH Terms] OR "Medical Device Legislation"[MeSH Terms] OR "Drug and Narcotic Control"[MeSH Terms] OR "International Health Regulations"[MeSH Terms]) OR (regulation*[Text Word]) OR (recommendation*[Text Word]) OR (legislation*[Text Word]) OR (polic*[Text Word]) OR (response*[Text Word]) OR (effort*[Text Word]) OR ("preventive measure*"[Text Word]) OR (combat*[Text Word]) OR ("infodemic management"[Text Word]) OR (debunk*[Text Word]) OR (solution*[Text Word]))	((("Health Literacy"[Mesh]) OR ("Communication"[Mesh]) OR "False information"[Text Word]) OR ("Incorrect information"[Text Word]) OR ("Inaccurate information"[Text Word]) OR (misinformation[Text Word]) OR (disinformation[Text Word]) OR (infodem*[Text Word]) OR ("false science"[Text Word]) OR ("misleading information"[Text Word]) OR ("fake news"[Text Word]) OR ("malinformation"[Text Word]) OR ("health washing"[Text Word]) OR ("unfair information"[Text Word]) OR ("Digital health literacy"[Text Words]) OR (Health literacy[Text Word]) OR ("information literacy"[Text Word]))		(("Social Media"[Mesh]) OR ("Media Exposure"[Mesh]) OR (TikTok[Text Word]) OR (Instagram[Text Word]) OR (Facebook[Text Word]) OR (Twitter[Text Word]) OR (Youtube[Text Word]) OR ("Snapchat"[Text Word]) OR ("Social media"[Text Word]))

Exemplar search in PubMed/MEDLINE

(((("social control, formal"[MeSH Terms] OR "Government Regulation"[MeSH Terms] OR "Medical Device Legislation"[MeSH Terms] OR "Drug and Narcotic Control"[MeSH Terms] OR "International Health Regulations"[MeSH Terms]) OR (Regulation*[Text Word]) OR (recommendation*[Text Word]) OR (legislation*[Text Word]) OR (polic*[Text Word]) OR (Response*[Text Word]) OR (Effort*[Text Word]) OR ("preventive measure*"[Text Word]) OR (combat*[Text Word]) OR ("infodemic management"[Text Word]) OR (debunk*[Text Word]) OR (solution*[Text Word]))) AND ((("False information"[Text Word]) OR ("Incorrect information"[Text Word]) OR ("Inaccurate information"[Text Word])OR (misinformation[Text Word]) OR (disinformation[Text Word]) OR (infodem*[Text Word]) OR ("false science"[Text Word]) OR ("misleading information"[Text Word]) OR ("fake news"[Text Word]) OR ("health washing"[Text Word]) OR ("malinformation"[Text Word]) OR ("Communication"[Mesh]) OR ("health literacy"[MeSH Terms]) OR (health literacy[Text Word]) OR ("Health Literacy"[Mesh])))) AND ((("Social Media"[Mesh]) OR ("Media Exposure"[Mesh]) OR (Tiktok[Text Word]) OR (Instagram[Text Word]) OR (Facebook[Text Word]) OR (Twitter[Text Word]) OR (Youtube[Text Word]) OR ("Snapchat"[Text Word]) OR ("Social media"[Text Word])))

Supplementary file 2: Supporting evidence from peer-reviewed literature

Study ID	Study design	Study funding sources	Conflicts of interest	Concept and definition
Alsaad 2024	Single-arm, pre-post intervention design (N=483)	King Abdullah International Medical Research Center	None declared	Misinformation: "(...)health misinformation is defined as any health-related factual claim that is false according to recent scientific evidence"
Au 2021	Online experiment (N=363)	None declared.	None declared	Misinformation: "From an academic perspective, online misinformation may be understood as any wrong information on the Internet (…)"
Braga, 2025	Machine learning for analysing Google cloud and Twitter API data	Study funding sources FCT (Fundação para a Ciência e Tecnologia): 00319. R&D Project: PTDC/SAU-ENF/2584/2021.	None declared	Misinformation: "Health misinformation involves the circulation of false or misleading information about health, shared without intent to cause harm(…)"
Bode 2018	Three-condition between-subjects experiment embedded in an online survey (N=136)	None declared	None declared	Misinformation: "(...)Misinformation is defined as“casesin which people’s beliefs about factual matters are not supported by clear evidence and expert opinion”(...)"
Brooks 2023	Collaborative and integrated development of framework for virtual center. Effects not tested in formal design.	None declared	None declared	Malinformation: Information that is based on reality, used to inflict harm on a person, organisation or country. Disinformation: Information that is false and deliberately created to harm a person, social group, organisation or country. Misinformation: Information that is false, but not created with the intention of causing harm.
Byrne 2024	Development of online platform. Effects not tested in formal design.	Health Research Board, Health Service Executive and the College of Medicine, Nursing and Health Sciences, University of Galway	None declared	Unreliable health information: N/A
DiSotto 2022	Comparing different social media features from publicly-available data sets using machine learning algorithms and the implementation of diverse networks.	None declared	None declared	Misinformation: Non-genuine health information: “a health-related claim of fact that is currently false due to a lack of scientific evidence” . "(...)no distinction between false information generated with the intent or not to cause harm."
Elhariry, 2024	Prospective, mixed-method study in collaboration with experts and patients develop content for social media. Evaluation of metrics of content (N=21 videos)	Society for Endocrinology	None declared	Misinformation: N/A, reffered to as: "...misleading or false information..."

Erim, 2025	Survey study evaluating the 3-day training programme (N=25 participants)	"This research was conducted as part of the project funded by Bill and Melinda Gates Foundation"	None declared	Misinformation: N/A
Fridman, 2025	Experiment employing predictive modelling derived from literature (N=88 papers) and tested on social media posts (N=45791)	North Carolina Translational Research and Clinical Science Institute, Pilot Award Spring 2022	N/A	Misinformation: "(…) information that promoted cancer treatment that was known as ineffective or information that suggested cancer causes not supported by current scientific evidence"
Garrett 2019	A five-step Delphi approach, comprised of a multidisciplinary panel of health professionals. Effects not tested in formal design.	University of British Columbia Hampton, Fund Research Grant in the Social Sciences	N/A	Internet Health Scams: "Nostrum sales—when products are sold based on exaggerated claims or falsehoods—and the use of mass media to facilitate scams (...)"
Gesser-Edelsburg 2018	A controlled experiment with participants divided randomly into two conditions (N=243)	None declared.	None declared	Misinformation (deficient information) or disinformation (intentionally false information).
Ku, 2025	2 × 2 experiment design with a control group (N = 274)	This work was supported by the General Research Fund of the Hong Kong Research Grant Council (grant number 12615922)	None declared	Misinformation: "Misinformation, defined as false or inaccurate information that contradicts available evidence..."
Lazard, 2025	Randomised longitudinal, within-persons online experiment with simulated social media platform (N=90)	This project was supported by UNC Center for Health Innovation's Innovation Pilot Award program	None declared	Misinformation about cancer treatments was determined as treatments: "(...) not supported by current scientific consensus and did not include a viable mechanism for treatment (e.g., not currently in clinical trials)."
Leininger 2022	Development of online platform. Effects not tested in formal design.	N/A	N/A	Misinformation: N/A but referred to as false, lies, harmful information
McPhedran 2023	Online experiment (N= 2430)	N/A	None declared	Misinformation: "(...) Inaccurate and sometimes deleterious information"
Mende 2023	Development of model. Effects not tested in formal design	Transformative Consumer Research grant	None declared	Misinformation: information introduced as true but recognised as erroneous, incorrect or misleading later. Shared with no intention to do harm. Disinformation: deceitful information distributed for propagandistic goals that later is recognised as inaccurate. False information distributed purposely/with intentional effort.
Nazarnia 2023	Quasi-randomised design (N=100)	This study was supported by the Faculty of Medical Sciences at Tarbiat Modares University in Tehran, Iran. This research received no external funding.	None declared	Mis- and disinformation: N/A

Ozturk, 2015	Online experiment (N= 259)	The National Science Foundation under Grant No. IIS-1138658 and Grant No. BCS-1244742	N/A	Health myths/rumors: N/A but described as either unverified or false according to health professionals
Sun, 2025	Factorial design experiment (N = 754).	"The author(s) reported there is no funding associated with the work featured in this article"	None declared	Misinformation: N/A
Upadhyay 2023	Experimental evaluation of model of public data sets	The European Union's Horizon 2020 Research and Innovation Programme under the Marie Skłodowska-Curie Grant Agreement No 860721.	N/A	Misinformation: "a health-related claim that is based on anecdotal evidence, false, or misleading owing to the lack of existing scientific knowledge". "In the literature, the concepts of disinformation, understood as false information propagated with malicious intent, misinformation, understood as false information generated without malicious intentions, and malinformation, understood as genuine information shared to cause damage, often moving information destined to remain private in the public sphere, have been introduced. The definition of health misinformation that we report and employ in this article, makes no distinction between false information generated with the intent or not to cause harm."
Vraga 2017	Online experiment embedded in survey (N=1348)	None declared	None declared	Misinformation: "(...) we use a commonly cited definition originated by Nyhan and Reifler (2010): "Cases in which people's beliefs about factual matters are not supported by clear evidence and expert opinion""
Vraga 2018	Online experiment embedded in survey (N=271)	N/A	None declared	Misinformation: "Worries about misinformation (e.g., false information) have been primarily focused on the difficulty of correcting the misperceptions – which we define as individual 'beliefs about factual matters [that] are not supported by clear evidence and expert opinion' (Nyhan & Reifler, 2010, p. 305) – that arise from acceptance of the misinformation."
Vraga 2019	Online experiment embedded in survey (N=406)	Department of Communication at George Mason University	None declared	Misinformation: N/A
Vraga 2022a	Online randomised experiment embedded in survey (N=916)	Page and Johnson Legacy Scholar Grant #2018FN004 from Pennsylvania State University	N/A	Misinformation: Mention of "fake news" and "misleading citizens"

Vraga 2022b	Online experiment embedded in survey (N=1348)	Page and Johnson Legacy, Scholar Grant from Pennsylvania State University and the University of Minnesota	N/A	Misinformation: False health information or health myths
Vraga 2025	Online experimental embedded in survey (N=1431)	"The author(s) received no financial support for the research, authorship, and/or publication of this article"	None declared	Misinformation: False information
